# Association between Normal Weight Obesity and Skeletal Muscle Mass Index in Female University Students with Past Exercise Habituation

**DOI:** 10.3390/jfmk7040092

**Published:** 2022-10-21

**Authors:** Kazushige Oshita, Ryota Myotsuzono, Tomoki Tashiro

**Affiliations:** 1 Department of Human Information Engineering, Okayama Prefectural University, 111 Kuboki, Soja 719-1197, Japan; 2 Department of Sport Science, Kyushu Kyoritsu University, 1-8, Jiyugaoka, Yahatanishi-ku, Kitakyushu 807-8585, Japan

**Keywords:** physical activity level, body mass index, body weight, body fat, sarcopenia

## Abstract

This study investigated the relationship between normal weight obesity (NW-O) and skeletal muscle mass index (SMI) in 120 female university students who participated in sporting activities during junior and senior high school. The current physical activity level (PAL) was estimated by a factorial method using 24-h physical activity recall. The body mass index (BMI) of the participants ranged from 18.5 to 24.9 kg/m^2^; their body fat (BF) was classified as NW-O if above the 75th percentile (28.9% BF), normal weight and lean (NW-L) if below the 25th percentile value (21.0%BF), and all others were normal weight (NW). PAL was significantly lower in NW-O than in NW-L. SMI was significantly lower in NW-O than in NW and NW-L, and 60% of NW-O had Low-SMI (<6.3 kg/m^2^). Although lower limb muscle mass was significantly lower in NW-O than in NW and NW-L, no significant differences were found in the upper limbs. These results suggest that the current PAL is associated with NW-O, and NW-O is associated with a lower SMI, even in young females with past exercise habits. Therefore, the prevention of NW-O in young females is important for the prevention of not only lifestyle-related diseases, but also future sarcopenia.

## 1. Introduction

Age-related decline in severe skeletal muscle mass and muscle strength and/or function in older people is termed “sarcopenia” [1]. To prevent future sarcopenia, it is important not only to maintain muscle mass and strength in middle age and minimize their decline in old age, but also to improve the peak values achieved in early adulthood [2,3]. Regarding muscle mass, the effectiveness of exercise interventions in frail, sedentary, community-dwelling older adults has been reported to not consistently improve [4]. Therefore, the enhancement of peak muscle mass in early adulthood is important for the prevention of sarcopenia. The “skeletal muscle mass index” (SMI), calculated from the sum of appendicular muscle mass (AMM), is used to assess muscle mass in the diagnosis of sarcopenia [5,6,7]. Since a high proportion of young Japanese females are underweight [8,9], the relationship between physical activity and/or nutritional intake and SMI has been reported in young females to prevent future sarcopenia [10,11]. Previous reports have shown that even among young females who regularly exercised in the past, approximately half of those with a low current physical activity level (PAL) have low muscle mass or a tendency toward low muscle mass according to the diagnostic criteria for sarcopenia [10]. Therefore, current lifestyles are important for maintaining sufficient muscle mass, even in young populations and regardless of past exercise habits.

However, many previous reports have been published on body composition from young to old regarding “normal weight obesity” (NW-O). NW-O is defined as a state of high body fat despite a body mass index (BMI; calculated from height and weight) in the normal range, and the risk of cardiometabolic dysregulation and systemic inflammation has been reported [12,13,14,15,16]. Having a high body fat content, even with body weight in the normal range, results in a low fat-free mass, including skeletal muscles. Previous studies have reported that NW-O in young females has lower muscle mass in the trunk and lower limbs compared to non-obese individuals [17]. However, the specific impact of this lower muscle mass on health in young females is not known. Assessing the muscle mass of NW-O individuals with the SMI value will give a specific reference to how serious it is, as it could be compared with the sarcopenia cut-off values.

In a recent review article [16], some previous studies have reported that NW-O is associated with a sedentary lifestyle or lower physical activity. Further, lower current PAL is associated with lower SMI in young females who regularly practiced sports in the past, suggesting that current PAL is important to prevent future sarcopenia, regardless of past exercise habits [10]. Based on these studies, we hypothesize that current lower PAL is associated with NW-O and that NW-O individuals are more likely to have lower muscle mass in the sarcopenia diagnostic criteria. However, previous studies on the relationship between current PAL and SMI [10] have focused only on SMI. Although body fat percentage (%BF) was not significantly differentiated by the current PAL in that study [10], the relationship between BMI or NW-O and the current PAL was unclear. If NW-O is also associated with a low SMI in young females, prevention of NW-O might be important not only to prevent lifestyle-related diseases and/or metabolic syndrome, but also for sarcopenia in the future. Therefore, the present study aimed to investigate the relationship between NW-O and SMI based on data from a previous study that reported the relationship between current PAL and SMI in young females with past exercise habits [10].

## 2. Methods

### 2.1. Participants

The present study used data from a previous study [10]. The participants were 120 female university students who participated in athletic club activities as athletes in both junior and senior high school, and whose current PAL was up to 2.2 (i.e., up to a high (III) PAL in the dietary reference intakes for Japanese (DRI-J) [18]).

All participants were informed verbally and in writing beforehand that their responses to the questionnaire would be anonymous and used only for research purposes. They were also informed that the data obtained would be published after statistical analysis so that no individuals could be identified. The survey was conducted only when the participants accepted these terms, and a written informed consent form was obtained. The study was approved by the Ethics Committee of Kyushu Kyoritsu University (No. 2018-15).

### 2.2. Estimation of PAL Using the Factorial Method

Daily activities on weekdays in the last month were investigated using a questionnaire-based 24 h of physical activity recall in which a day was divided into 288 periods of 5 min each.

PAL was assessed by calculating the daily average from the product of the following activity indices (energy expenditure expressed as a multiple of the basal metabolic rate) and the duration of each activity: 1.0 for sleeping; 1.1 for lying down; 1.5 for sitting; 1.7 for light work in a standing position; 2.5 for slow-moving and housework; 4.0 for activities of daily living and work that can be sustained to some extent; 5.0 for activities of daily living and work of high intensity requiring a rest period; and exercise/sport activity (1.7 for rest periods, 4.0 for low intensity, 5.0 for low-to-medium intensity, 6.5 for medium intensity, and 8.0 for high-intensity activity). These details are described in a previous study [10].

### 2.3. Assessment of Muscle Mass and NW-O

Weight and body composition were measured using a dual-frequency bioelectrical impedance analysis (BIA) body composition analyzer (RD-E04, Tanita Corporation). The palms and soles of the participants were wiped with alcohol-free wet wipes to remove dirt and moisten the electrode contact areas. The participants then stood on the electrode part of the analyzer, while holding the hand electrodes with both palms, and their body composition was measured. The values measured with this analyzer in a young population demonstrated significantly linear relationships with the values measured with the multi-frequency BIA analyzer used in studies on sarcopenia, with a coefficient of determination of 0.95 or higher for BF, muscle mass, and AMM, and the slopes of the regression line ranging from 1.002 to 1.136 with intercepts of –1.498 to 0.570 [19].

BMI was calculated by dividing weight (kg) by the square of height (m), and SMI was calculated by dividing AMM (kg) by the square of height (m). For the cut-off value of SMI for sarcopenia, Janssen et al. [20] established two criteria for low muscle mass: class 1, less than the mean SMI-1 standard deviation (SD) in the young population; class 2, less than the mean SMI-2 SD. Since class 2 corresponds to low muscle mass in the diagnosis of sarcopenia [5,6,7], it is referred to as “Low-MM” in this study. Class 1 was referred to as “Low-SMI” in this study because it is believed to be a preliminary cluster of low muscle mass in the diagnosis of sarcopenia.

Although a recent review article has used the term “NW-O” to describe any group of individuals who were characterized using BMI and a body fat percent cutoff, it was noted that no definitive cut-off value of %BF for NW-O has been specified [16]. In a study investigating the relationship between NW-O and lifestyle in Japanese female university students [21], the criteria for NW-O as BMI in the normal range (18.5–24.9 kg/m^2^) and %BF above the 75th percentile of the entire participants. Therefore, participants in the present study whose BMI ranged from 18.5 to 24.9 kg/m^2^ and whose %BF was above the 75th percentile value (28.9%BF) for the entire participant population were classified as NW-O. This %BF value is slightly higher than in the previous study (27.0%BF) [21]. A %BF below the 25th percentile value (21.0%BF) was classified as normal weight and lean (NW-L), and all others were simply normal weight (NW). Those with a BMI <18.5 kg/m^2^ and ≥25.0 kg/m^2^ were defined as underweight (UW) and overweight (OW), respectively.

### 2.4. Statistical Analysis

The differences in mean values between NW-O, NW, and NW-L for PAL, height, weight, SMI, and muscle mass (upper limb, lower limb, and AMM) were compared using the Kruskal–Wallis test, and multiple comparisons using Scheffe’s method were performed if significant differences were found. Cohen’s *d* values were calculated as effect sizes. The *χ*^2^ test was used to compare the proportion of Low-SMI in NW-O, NW, and NW-L, and residual analysis was performed if a significant difference was found.

These analyses were performed using the JSTAT (ver.12.5, Japan) and js-STAR (ver. 9.8.6j, Japan) software. The level of statistical significance was set at <5%. The effect size was established as small for *d* < 0.2, moderate for 0.2 ≤ *d* < 0.8, and large for *d* ≤ 0.8.

## 3. Results

The means and SDs of each variable for all participants are presented in Table 1. Twenty-eight participants had a Low-SMI, of which four had a Low-MM. Four participants had UW, 27 had NW-L, 59 had NW, 25 had NW-O, and five had OW.

The means and SDs of NW-L, NW, and NW-O for each variable are presented in Table 2. No significant main effects were found between the groups for height and upper limb muscle mass, and the effect sizes were less than moderate. Body weight and SMI were significantly higher in NW-O than in NW-L and NW, with moderate- or large-size effects. Lower limb muscle mass and AMM were significantly lower in the NW-O group than in the NW-L and NW groups, and the effect sizes were large. They were also significantly lower in the NW group than in the NW-L group and had moderate-sized effects. PAL was significantly lower in the NW-O group than in the NW-L group and had a large effect. The BMI, %BF, PAL, and SMI of UW (n = 4) were 17.8 ± 0.5 kg/m^2^, 20.7 ± 4.3%, 1.5 ± 0.1, and 5.9 ± 0.3 kg/m^2^, respectively. Furthermore, the BMI, %BF, PAL, and SMI of OW (n = 5) were 25.8 ± 0.6 kg/m^2^, 33.9 ± 2.8%, 1.9 ± 0.2, and 7.2 ± 0.8 kg/m^2^, respectively.

**Figure 1:**
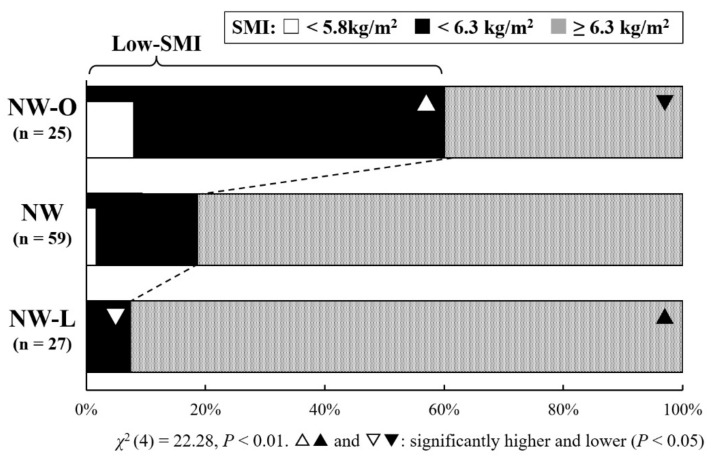
The proportion of Low-SMI in NW-L, NW, and NW-O.

The proportions of Low-SMI in NW-L, NW, and NW-O are shown in Figure 1. The proportion of Low-SMI was 60.0% (15 participants) in NW-O, 18.6% (11 participants) in NW, and 7.4% (2 participants) in NW-L (*χ*^2^ (4) = 22.28, *p* < 0.01), with a significantly higher proportion in NW-O. Among these, Low-MM was found in two NW-O and one NW but not in NW-L. All UW participants had Low-SMI, including one participant with Low-MM, and none of the OW participants had Low-SMI.

## 4. Discussion

This study investigated the relationship between NW-O and SMI in young females with past exercise habits. We found that the current PAL was significantly lower in NW-O than in NW-L. The SMI was significantly lower in NW-O than in NW or NW-L, with 60% of NW-O having Low-SMI (<6.3 kg/m^2^). Lower limb muscle mass was significantly lower in NW-O than in NW or NW-L, and no significant differences were found in the upper limb.

In the present study, PAL was significantly lower in NW-O than in NW-L. Previous studies have also reported that NW-O is associated with low PAL or a sedentary lifestyle [16]. The results of this study indicate that a lower current PAL is associated with higher body fat despite a normal body weight range, even if the individual had an exercise habit in the past. However, the average PAL of NW-O is 1.7, which is almost the lower limit of normal PAL according to the DRI-J [18]. The daily activities in this study were investigated on an average weekday. Since the participants were university students, they may have maintained a normal PAL level due to commuting to campus, taking classes, and so on. Studies investigating the physical activity of university students report that they take fewer steps [22], lower PAL (average physical activity and moderate to vigorous physical activity) and spend more sedentary periods [23] on weekends than on weekdays. Furthermore, although no significant difference was found regarding the average number of steps between the sexes on weekdays, female students had lesser average steps on weekend days compared to male students [22]. Therefore, an additional investigation of PAL on holidays, excluding the influence of social activities, is necessary. Furthermore, fat-free mass is lower in cases with lower total energy and protein intake, even with high PAL [11]. Therefore, future research should include the nutritional intake status.

The SMI was significantly lower in NW-O than in NW or NW-L. As NW-O has normal body weight but a high %BF, it is natural that they would have a lower SMI. However, surprisingly, 60% of the participants with NW-O had low-SMI which means class 1 sarcopenia according to the SMI criteria of Janssen et al. [20]. Sarcopenia is diagnosed in people aged ≥65 years and is accompanied by a lower SMI and lower muscle strength or function [5,6,7]. However, if approximately 20-year-old NW-O participants can maintain their current SMI for a further 45 years, they will be diagnosed with sarcopenia or pre-sarcopenia in cases of accompanying low grip strength or gait speed. Therefore, although NW-O has been widely reported from the perspective of lifestyle-related diseases and/or metabolic syndrome, the results of this study suggest that it is also important for the future prevention of musculoskeletal or locomotive disorders and sarcopenia. As this study focused on NW-O, the numbers of UW and OW participants were small and were not compared to NW-O. Although only four participants had UW, all had a Low-SMI. Therefore, not only is NW-O associated with Low-SMI in young females, but underweight is also at risk of leading to Low-SMI.

Lower limb muscle mass was significantly lower in NW-O than in NW or NW-L, with no significant differences in the upper limbs. This is consistent with the results of a previous study of Japanese participants in the same age group [17]. NW-O individuals in this study also had significantly lower SMI, which means that low SMI is mainly related to low lower limb muscle mass. Although BMI in NW-O individuals are within the normal range (18.5–24.9 kg/m^2^), body weight was significantly higher in NW-O than in NW or NW-L. This higher body weight may reflect a higher %BF. Therefore, NW-O individuals need to support a relatively heavier body weight with lower leg muscles. In older people, the combination of sarcopenia and obesity is called “sarcopenic-obesity” [24,25], which is associated with poorer gait and balance ability than sarcopenia alone and is more likely to cause physical disability and falls [24]. Since the participants in this study were young females, there may not be an immediate impact on daily activities. However, NW-O has also been reported to be associated with lower physical fitness [12,15,16,26]. Therefore, NW-O in the younger generation may impair activities of daily living in the near future.

NW-L participants had a higher current PAL, indicating that they consistently participated in sports or exercise activities from the past to the present. Therefore, their lower limb muscle mass and AMM were higher than those of the NW group. However, the average %BF was 18.5%. Although body fat is necessary to maintain a normal ovulatory menstrual cycle in women [27,28], some NW-L participants may have insufficient %BF. NW-L had a higher SMI, which is advantageous for the future prevention of sarcopenia. However, maintaining adequate body fat through sufficient dietary intake is important for women’s health.

Although the present study investigated the relationship between NW-O and SMI in young females with past exercise habits, it has several limitations. First, PAL was evaluated using a questionnaire-based survey. Some previous reports suggest that PAL by questionnaire is not related to NW-O [16]. The actual measurement of PAL, including weekdays and holidays, is necessary for future research. Second, this is a cross-sectional study. Although NW-O was related to current PAL, even in young females with a past exercise habit changes in body composition due to changes in PAL are unclear. Therefore, longitudinal studies are required in the future. Finally, it is necessary to consider not only PAL but also other lifestyle habits. For example, young females with high PAL but lower total energy or protein intake have been reported to have a lower SMI and higher %BF [11]. Therefore, future studies should include other lifestyle factors such as diet.

## 5. Conclusions

This study investigated the relationship between NW-O and SMI in female university students who participated in sports activities during junior and senior high school. Our results showed that the current PAL is associated with NW-O and NW-O is associated with lower SMI, even in young females with past exercise habits. Furthermore, all UW participants with Low-SMI indicated that being underweight in young females was accompanied by a lower SMI. These results suggest that the prevention of NW-O in young females is important not only for the prevention of lifestyle-related diseases and/or metabolic syndrome but also for the future prevention of musculoskeletal or locomotor disorders and sarcopenia.

## Figures and Tables

**Table 1:** Means and standard deviations of each variable of the entire participants.

Height (cm)		159.5	±	4.8
Weight (kg)		55.3	±	5.3
BMI (kg/m^2^)		21.7	±	1.8
%BF (%)		24.9	±	5.0
SMI (kg/m^2^)		7.12	±	0.93
Muscle mass	Upper limb (kg)	3.4	±	0.4
	Lower limb (kg)	14.7	±	2.2
	Appendicular (kg)	18.1	±	2.6
PAL		1.8	±	0.2

**Table 2:** Means and standard deviations of NW-L, NW and NW-O for each variable.

		Body Weight—Boby Composition											Effect Size (Cohen’s *d*)		
		NW-L			NW				NW-O				NW-L vs. NW	NW-L vs. NW-O	NW vs. NW-O
		BMI = 20.4 ± 1.2			BMI = 21.8 ± 1.3				BMI = 22.8 ± 1.2						
		%BF = 18.5 ± 1.8			%BF = 24.8 ± 2.4				%BF = 30.7 ± 1.3						
Height (cm)		160.6	±	4.5	158.9	±	5.1		159.4	±	4.9		0.4	0.3	0.1
Weight (kg) **		52.7	±	3.7	55.1	±	4.8		58.1	±	4.1	^†, ‡^	0.5	1.4	0.6
SMI (kg/m^2^) **		7.6	±	0.8	7.2	±	0.9		6.5	±	0.7	^†, ‡^	0.5	1.5	0.9
Muscle mass	Upper limb (kg)	3.5	±	0.4	3.4	±	0.4		3.3	±	0.4		0.4	0.6	0.2
	Lower limb (kg) **	16.1	±	1.8	14.9	±	2.2	^†^	13.1	±	1.5	^†, ‡^	0.6	1.8	0.9
	Appendicular (kg) **	19.7	±	2.2	18.3	±	2.6	^†^	16.5	±	1.8	^†, ‡^	0.6	1.6	0.8
PAL *		1.9	±	0.2	1.8	±	0.2		1.7	±	0.2	^†^	0.6	1.0	0.3

* and **; *p* < 0.05 and 0.01 (Kruskal–Wallis test). ^†^ and ^‡^ *p* < 0.05 (vs. NW-L and NW, Scheffe’s test).

## Data Availability

The datasets generated and analyzed during the current study are available from the corresponding author on reasonable request.
